# Advancing Therapeutic Drug Monitoring for Oral Targeted Anticancer Drugs: From Hospital‐Based Towards Home‐Sampling

**DOI:** 10.1002/bmc.70056

**Published:** 2025-03-14

**Authors:** Marinda Meertens, Hilde Rosing, Neeltje Steeghs, Jos H. Beijnen, Alwin D. R. Huitema

**Affiliations:** ^1^ Department of Pharmacy & Pharmacology The Netherlands Cancer Institute – Antoni van Leeuwenhoek Hospital Amsterdam The Netherlands; ^2^ Department of Medical Oncology The Netherlands Cancer Institute, Antoni van Leeuwenhoek Amsterdam The Netherlands; ^3^ Department of Medical Oncology Utrecht University Medical Centre, Utrecht University Utrecht The Netherlands; ^4^ Division of Pharmacoepidemiology and Clinical Pharmacology, Utrecht Institute for Pharmaceutical Sciences Utrecht University Utrecht The Netherlands; ^5^ Department of Clinical Pharmacy, University Medical Center Utrecht Utrecht University Utrecht The Netherlands; ^6^ Department of Pharmacology Princess Máxima Center for Pediatric Oncology Utrecht The Netherlands

**Keywords:** dried blood spots, home‐sampling, LC‐MS/MS, oral targeted anticancer therapies, therapeutic drug monitoring, volumetric absorptive microsampling

## Abstract

Home‐sampling for therapeutic drug monitoring (TDM) for oral targeted anticancer drugs offers a promising alternative to traditional hospital‐based sampling methods, though it presents challenges. This review aims to summarize the state‐of‐the‐art of home‐sampling methods for TDM and evaluates the analytical and clinical validation challenges. A comprehensive search was conducted across Embase, Medline, and Scopus. Eligible articles described analytical and/or clinical validation of home‐sampling methods for oral targeted anticancer drugs. ASReview was used to process unique references and to identify relevant studies. Of the 39 included articles, 32 detailed on analytical validation experiments, while 27 covered clinical validation experiments. Dried blood spot and volumetric absorptive microsampling were the primary sampling methods. Key challenges were ensuring robust sample collection, sample pretreatment, hematocrit effects, and sample stability, which were generally thoroughly investigated. Clinical validation yielded promising results for most analytes, although external validation remains crucial for confirming reliability. Home‐sampling methods for TDM of oral targeted anticancer drugs show promising results for clinical implementation. Methods for well‐studied drugs may be clinically implemented immediately, while others require further external validation. Future research should address device‐specific challenges and assess patient feasibility to facilitate the routine use of home‐sampling in clinical practice.

## 1 Introduction

Therapeutic drug monitoring (TDM) is a practical tool for personalizing oral targeted anticancer therapy. For several agents, TDM has the potential to enhance efficacy and to reduce toxicity, leading to recommendations for its routine use (van der Kleij et al. 2023). In specific scenarios such as bariatric surgery or drug–drug interactions, TDM may provide benefits irrespective of whether it is routinely recommended (Lau et al. 2024; Le Louedec et al. 2023).

Home‐sampling for TDM presents a promising alternative to traditional hospital‐based methods, offering several advantages. A hospital‐based procedure necessitates presence of the patient at a medical facility for venepuncture. The home‐sampling approach is already common in fields like organ transplant patients, and its application in the field of oncology could potentially lead to significant advancements (Brunet et al. 2019). For patients, it is less invasive as it requires smaller blood volumes and enables rapid dose adjustments without the need for (expensive) hospital visits. The reliability could also be enhanced through the possibility of drawing trough levels at home, which is not always feasible in the hospital setting due to the random timing of hospital visits. Analytically, home‐sampling methods offer benefits like improved stability due to the dried form, room temperature storage avoiding freeze/thaw cycles, and relatively simple sample preparation. This could potentially reduce costs by decreasing the need for trained staff for venepuncture, eliminating the need for dry ice transport and avoiding necessity of plasma storage at −70 °C (Verougstraete et al. 2022).

Currently, various assays and devices are available for home‐sampling, with dried blood spots (DBS) and volumetric absorptive microsampling (VAMS) being the most commonly reported in the literature (Delahaye et al. 2021). Some methods such as dried plasma spots, where plasma is aliquoted onto DBS filter paper, are not suitable for home use. After selection of a convenient sampling method, the following critical step is the development of a robust analytical method. This requires comprehensive validation including standard analytical validation experiments as well as specific tests for home‐sampling devices, such as extraction recovery and hematocrit (Hct) influence assessments, among additional tests (Capiau et al. 2019; European Medicines Agency 2011).

Subsequently after the establishment of a validated analytical method, clinical validation must be conducted (European Medicines Agency 2011). Despite having an operational robust analytical method, successful clinical validation is not guaranteed (Zimmermann et al. 2023). When whole blood results correlate well with plasma levels, the process is relatively straightforward. However, in cases where they do not, a conversion method must be developed for accurate clinical interpretation. Different strategies for clinical interpretation include correcting for Hct levels or employing a conversion factor (Iacuzzi et al. 2021). Even after successful analytical and clinical validations, it remains essential that patients correctly collect samples to ensure reliable results, which requires further evaluation in daily practice (Boffel et al. 2024).

While the use of home‐sampling offers numerous benefits, it also presents several challenges. The aim of this review is to summarize the current state‐of‐the‐art of home‐sampling methods for TDM of oral targeted anticancer drugs, to evaluate the entire process from analytical and clinical validation to practical implementation in clinical practice.

## 2 Methods

### 2.1 Search Strategy

**FIGURE 1:**
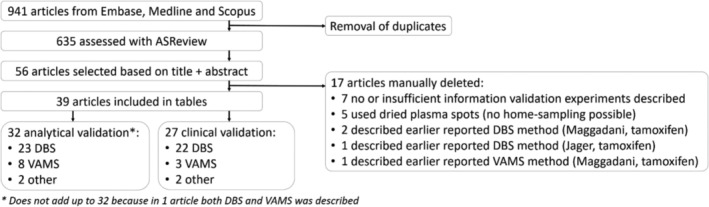
Flowchart article filtering and inclusion.

A comprehensive search was conducted across Embase, Medline, and Scopus, yielding a total of 941 references. After removing duplicates, 635 unique references remained (Figure 1). The search strategy employed is detailed in the Supplemental material.

### 2.2 Article Filtering and Inclusion and Exclusion Criteria

The unique references were processed using ASReview, an AI‐assisted tool. ASReview was used in its default mode following the initial upload of five relevant and five non‐relevant articles. Criteria for completion required screening at least 25% of the articles and identifying 10% of all articles as non‐relevant in one row (i.e., respectively 159 articles and 64 non‐relevant in this case) (van de Schoot et al. 2021). The relevant records based on title and abstract screening underwent further evaluation based on full‐text analysis to assess their suitability for inclusion.

Articles were considered eligible if they described a home‐sampling method for analyzing one of the drugs of interests in oncology, including oral targeted therapies and oral anti‐hormone therapies (see Supplemental material). Studies focusing on either analytical validation and/or clinical validation experiments were included. Exclusions comprised articles concerning microsampling methods applied in non‐human species, reviews, insufficient detail provided for analytical and/or clinical validation, and studies specific for everolimus in transplantation populations.

### 2.3 Data Collection

Data regarding analytical and clinical validation experiments were collected and summarized in tables. For the analytical validation, information was collected on the technique used, sample volumes, sample preparation procedures, analytical ranges, extraction recovery rates, stability, and critical factors. Only the circumstances outside the hospital were considered for stability, referred to as transport stability. For the clinical validation, data were collected on the number of collected samples and patients involved, comparisons between the different matrices and the tested conversion methods including their predictive performance.

## 3 Results and Discussion

### 3.1 Literature Search

Ultimately, 264 (42%) of the 635 articles were screened until 64 consecutive non‐relevant records were identified, marking the termination of screening after the last relevant article were found. Using this approach, 56 relevant records were selected based on title and abstract screening. These 56 articles were evaluated by full‐text analysis to assess their suitability for inclusion. These articles were then assessed for analytical and clinical validation experiments, as depicted in Figure 1. Out of these, five articles were excluded because they used dried plasma spots, which require centrifuging whole blood—a process unsuitable for the home‐sampling setting. Additionally, seven articles were excluded due to a lack of relevant information on analytical or clinical validation, or because only the application of a method was described without sufficient analytical details. One method appeared in duplicate across two journals—one local and one international; the local was excluded. Furthermore, two sampling methods (both from the same research group) were covered in multiple publications: one method was reported three times for DBS and one for VAMS, and another combination article described both methods together. The combination article was selected for inclusion, while the other four articles were excluded. Ultimately, 39 articles were included in this review. Of these, 32 articles detailed analytical validation experiments, while 27 reported (also) on clinical validation experiments. Specifically, 27 articles focused on DBS, 9 on VAMS, 1 on both DBS and VAMS, 1 on the Microsampling Wing and 1 on the Rhelise device. These four different sampling methods were first discussed.

#### 3.1.1 Dried Blood Spots

DBS sampling was the most reported home‐sampling method in our search. This technique uses paper cards, such as the widely used Whatman 903 protein saver, where a drop of whole blood is “spotted” onto the card. In case of home‐sampling, the volume of this drop of capillary whole blood is variable, therefore a fixed punch size is commonly used for quantification. The Whatman 903 protein saver cards are cellulose‐based, whereas other types of cards, such as DMPK‐A cards, include additional additives like Tris and SDS. Both Tris and SDS are surfactants that can denature proteins (Jager, Rosing, Schellens, and Beijnen 2014). In contrast, DMPK‐C cards are, similar to the Whatman 903 protein saver cards, not impregnated with additives and may be more suitable for preserving protein‐based molecules. Therefore, the choice of card type should be based on the properties of the drug. For instance, DMPK‐A cards, impregnated with Tris and SDS, provided better extraction for vemurafenib, a hydrophobic drug with poor aqueous solubility (Food and Drug Administration 2011; Nijenhuis et al. 2014). The volumetric DBS device hemaPEN was also reported. This device collects four DBS of 2.74 μL simultaneously, thus 10.96 μL in total (Deprez et al. 2019; Neto et al. 2018; Venkatesh et al. 2023). Another DBS device, the Hemaxis DB 10, collects the drop of blood via a micro‐channel, instantly absorbing it followed by spotting 10 μL onto DBS paper card, resulting in volumetric DBS samples (Canil et al. 2023; Leuthold et al. 2015).

#### 3.1.2 Volumetric Absorptive Microsampling

The VAMS device, branded as Mitra®, was the second most reported technique. VAMS uses a polymeric tip which absorbs a fixed volume of blood (10, 20, or 30 μL), which then can be removed from the stick for sample preparation. This sampling method addresses the limitations of conventional DBS, such as variable spot volume and homogeneity issues (Denniff and Spooner 2014).

#### 3.1.3 Liquid Microsampling Devices

Two other identified liquid microsampling devices were the Microsampling Wing (MSW) from Shimadzu, and the Rhelise kit from Redhot diagnostics, both using volumetric capillaries. The MSW, a small capillary with K_2_EDTA coating for anticoagulation, fills with 23 μL of blood and can be centrifuged to yield 5.6 μL of plasma by snapping of one part of the capillary (Hotta et al. 2021; Saito et al. 2022). The Rhelise kit, which fills with 50 μL of blood, mixes with a protein extraction liquid, precipitating the blood to maintain stability (Rehnmark et al. 2022).

#### 3.1.4 Data Collection

Information from the included articles on analytical and clinical validation experiments has been summarized in Tables 1 and 2, respectively. Both tables list the first author, technique, brand device, and drugs of interest. Table 1 focuses on analytical validation experiments, including sample volume, sample preparation, analytical range, overall extraction recovery, stability data, and critical factors like Hct. These critical steps will be discussed in detail in Section 3.2. Accuracy and precision were not included since all methods met the required criteria (European Medicines Agency 2011, 2022). All included analytical methods utilized liquid chromatography–tandem mass spectrometry for quantification. Accordingly, this review will focus on the analytical aspects inherent to this method. Table 2 summarizes the tested conversion methods and the predictive performance of those methods. Additionally, a column for remarks is included. These experiments will be discussed in Section 3.3.

**TABLE 1:** Validated chromatographic mass spectrometric methods using home‐sampling devices to support TDM.

First author (year)	Drugs	Technique; device	Sample volume	Sample preparation	Range (ng/mL)	Extraction recovery (CV%)	Transport and storage stability	Critical factors
Venkatesh et al. (2023)	Osimertinib	DBS; HemaPEN®	Volumetric 20 μL for 4 × 2.74 μL	Extraction with acetonitrile and IS working solution, while mixing	1–729	LLOQ, 92.9 (3.8) Low, 97.9 (5.5) Mid, 95.2 (7.3) High, 98.9 (5.6)	RT: 10 d; 4 °C: 72 h; −80 °C: 1 mo	Hct 0.30–0.60 no significant impact accuracy and precision
	AZ5104					LLOQ, 91.8 (4.2) Low, 100.9 (3.5) Mid, 96.6 (4.3) High, 101.2 (8.9)		
	AZ7550					LLOQ, 91.4 (8.2) Low, 103.8 (6.5) Mid, 95.4 (1.7) High, 103.1 (9.2)		
Canil et al. (2023)	Niraparib	DBS; Whatman 903 (calibrators and QCs) DBS; Hemaxis DB10 (patients)	Whatman 903: 8 mm punch–10 μL aliquotsHemaxis DB10: volumetric 10 μL	Extraction with IS working solution (methanol) while mixing.	60–3000	Low, 56 (10) Mid, 56 (6) High, 57 (5)	20 °C, <35% humidity: 219 d	Hct 0.29–0.45 no significant impact on accuracy and precision (Hct 0.25–0.55 did not meet requirements for accuracy)
	Olaparib				140–7000	Low, 93 (7) Mid, 94 (6) High, 92 (6)		
	Rucaparib				100–5000	Low, 66 (8) Mid, 68 (8) High, 66 (4)		
Zanchetta et al. (2023)	Lenvatinib	DBS; Whatman 31ETCHR and Whatman 903	8 mm punch–10 μL aliquots	Extraction with IS in methanol and 0.1% formic acid while mixing.	5–2000	Whatman 31ETCHR: Low, 85.8 (5.8) Mid, 77.2 (3.4) High, 76.6 (4.1) Whatman 903, Low, 82.7 (6.7) Mid, 77.3 (3.2) High, 80.1 (3.3)	All at low and high:−80 °C: 2 F/T cycles 50 °C: 4 d RT in paper envelope inside dryer: 98 d −80 °C in plastic envelope: 25 d;	5 μL and 10 μL spot volumes were underestimated with 3 mm punches, therefore 8 mm punch covering the whole spot.Hct 0.25–0.55 at 3 QC levels on both Whatman papers, all no significant impact on accuracy and precision.
Mukai et al. (2022)	Bosutinib	DBS; Whatman 903	Whole spot cut–40 μL aliquots	Extraction with acetonitrile/methanole (1/3 (v/v)) containing IS, while vortex mixing.	10–1000	Low, 105.5 (5.0) High, 81.8 (3.7)	40 °C and 90% RH: 72 h for all analytes RT: 12 weeks for all analytes, except ibrutinib 8 weeks.	Hct 0.20–0.50 at Low and High no significant impact on assay performance
	Dasatinib				2–200	Low, 63.6 (6.5) High, 71.7 (2.1)		
	Ibrutinib				4–400	Low, 89.8 (1.7) High, 81.4 (0.7)		
	Imatinib				40–4000	Low, 110.3 (1.5) High, 95.7 (2.3)		
	Nilotinib				40–4000	Low, 69.4 (2.5) High, 82.3 (0.6)		
	Ponatinib				2–200	Low, 72.8 (2.2) High, 76.2 (2.7)		
Zhang et al. (2022)	Anlotinib	DBS; Whatman 903	3 mm punch–15 μL aliquots	Extraction with 100 μL methanol with IS while vortex mixing, followed by sonication.	5–1000	Low, 64.27 (11.35) Mid, 59.33 (13.99) High, 64.68 (5.68)	Both at low and high: RT: 3 mo −80 °C: 3 mo	Hct 0.30–0.55, blood spot volume: 10, 20, 30 μL, and different punch sites all no significant impact on assay performance.
Braal et al. (2021)	Ribociclib	DBS; Whatman 903	6 mm punch–50 μL aliquots	Vortex mixing and sonication with IS in methanol	10–1000	Low, 72.0 (7.29) High, 64.8 (2.73)	At low and high, protected from light: controlled cabin 20 ± 5 °C RH 25%: 5 mo 3–7 °C: 5 mo RT: 5 mo	Hct 0.20–0.40 at High and Low no significant impact on assay performance.
Poetto et al. (2021)	Palbociclib	DBS; Whatman 31ETCHR	3 mm punch–20 μL aliquots	Extraction by 30 μL IS in methanol while mixing.	1–250	Low, 70.5 (10.4) Mid, 67.6 (5.3) High, 70.2 (2.6)	RT and −80 °C (3 F/T): 2.5 mo for all analytes;50 °C: 48 h for all analytes;	Hct 0.25–0.49 at QC low, mid and high, blood spot volume (5, 20 and 40 μL) and different punch sites, all had no significant impact on assay performance.
	Ribociclib				40–10,000	Low, 71.1 (11.3) Mid, 69.8 (7.0) High, 75.7 (5.2)		
	Letrozol				2–500	Low, 75.6 (3.8) Mid, 76.6 (2.2) High, 80.3 (3.8)		
Guo et al. (2021)	Bortezomib	DBS; Whatman 903	6 mm punch–50 μL aliquots	Extraction with methanol containing IS	0.2–20	Low, 97.5 (NR) Mid, 92.8 (NR) High, 91.0 (NR)	At Low, Mid, and High at:RT: 7 d; 4 °C: 14 d; −80 °C: 60 d;	Hct 0.20–0.50 no significant impact on assay performance.
Dillenburg Weiss et al. (2021)	Abiraterone	DBS; Whatman 903	2 × 8 mm punch–50 μL aliquots	Two punches cut in four pieces. Extraction with methanol containing IS. Incubation at 30 °C while shaking, evaporation at 60 °C.	1–400	Hct 0.20 Low, 84.6 Hct 0.20 High, 86.7 Hct 0.40 Low, 79.7 Hct 0.40 High, 72.6 Hct 0.60 Low, 77.8 Hct 0.60 High, 75.9	At Low and High:RT: 7 d; 2–8 °C: 7 d; 60 °C: 48 h;	Hct 0.20–0.50 no influence on accuracy and precision, Hct 0.60 did significant impact accuracy; Hct 0.20–0.60 no influence on recovery. Spot volume 20–60 μL no impact on accuracy.
	Delta(4)‐abiraterone				0.2–20	Hct 0.20 Low, 88.2 Hct 0.20 High, 99.7 Hct 0.40 Low, 89.6 Hct 0.40 High, 83.4 Hct 0.60 Low, 88.8 Hct 0.60 High, 89.6		
Iacuzzi et al. (2019)	Imatinib	DBS; Whatman ET31CHR	3 mm punch–20 μL aliquots	Extraction with 150 μL IS in 0.1% formic acid in methanol, while gently mixing	50–7500	Low, 74.8 (5.4) Mid, 77.7 (4.2) High, 80.5 (2.5)	RT: 16 mo	At low, mid, high: Hct 0.29–0.59; punch site and spot volumes 10–40 μL; all no significant impact on assay performance
	Norimatinib				10–1500	Low, 66.5 (7.7) Mid, 66.6 (4.6) High, 68.5 (2.1)		
Irie et al. (2018)	Gefitinib	DBS; Advantec qualitative filter paper no. 2, 125 g/m^2^	3 mm punch–10 μL aliquots	Extraction wit IS in methanol, while shaking	40–2400	Low, 104.9 (2.0) Mid, 96.3 (4.7) High, 95.7 (3.3)	At High, Mid, Low: RT and −20 °C: 5 mo; 40 °C: 24 h;	NR
Knapen et al. (2018)	Everolimus	DBS; Whatman 903	7.5 mm punch–30 μL aliquots	Extraction via sonication with IS in methanol/acetonitrile	3–75	74% (freshly prepared DBS); 81% (DBS samples stored 17 d at 2–8 °C)	−20 – −30 °C: 17 d 2–8 °C: 80 d 15–25 °C: 17 d	Hct 0.20–0.50 tested at 5, 20 and 40 ng/mL, assay performance no significant impact for Hct ≥ 0.25 for all concentrations; except at Hct 0.20 and Mid and High accuracy was >15% of nominal conc. 20–50 μL spot volumes and Hct 0.40 had minor impact. At Hct 0.20, spot volume had significant impact on accuracy.
Boons et al. (2017)	Nilotinib	DBS; Whatman FTA DMPK‐C	8 mm punch–40 μL aliquots	Extraction with methanol incl. IS, while shaking.	17–4100	87.6	At low and high: 2–8 °C: 7 mo;	Hct 0.25–0.50 at Low and High:no significant impact on accuracy.
Tré‐Hardy et al. (2016)	Tamoxifen	DBS; Whatman 903	3/16 in. (4.7625 mm) punch–50 μL aliquots	(Disks were cut in half) Extraction via sonication with IS in methanol, shaking and vortex mixing with small glass balls, evaporation with nitrogen at 50 °C.	1.7–500	Low, 95.2 (5.3) Mid, 96.3 (2.1) High, 95.2 (2.1)	NR	Hct 0.25–0.50 tested, for Hct ≥ 0.40 > 15% variation from the nominal value was observed, lower Hct had no impact on accuracy.
	4‐Hydroxy‐tamoxifen				0.6–500	Low, 83.8 (13.5) Mid, 95.6 (3.3) High, 91.8 (2.9)		
	Z‐endoxifen				3–500	Low, 88.3 (12.3) Mid, 88.9 (6.3) High, 95.9 (1.8)		
	N‐desmethyl tamoxifen				2–500	Low, 90.8 (8.2) Mid, 87.3 (6.8) High, 92.6 (6.3)		
Verheijen et al. (2016)	Pazopanib	DBS; Whatman 903	3 mm punch–15 μL aliquots	Vortex mixing and shaking with formic acid (99%), again vortex mixing and shaking with IS in methanol	1.000–50.000	Low, 97.6 (≤ 2.7) High, 103.7(≤ 2.7)	Ambient: 398 d;	All at Low and High: Punch site, spot volumes 10–30 μL and Hct 0.35–50.0 all no significant impact on accuracy and precision.
Antunes, Raymundo, de Oliveira, et al. (2015)	Tamoxifen	DBS; Whatman 903	10 mm punch–60 μL aliquots	(Two disks were used for extraction, cut in half.) Vortex mixing with IS in methanol, ultrasonication, evaporation (55 °C).	7.5–300	Low, 89.8 Mid, 91.3 High, 93.4	All at low and high: −20 °C, 25 °C and 45 °C: 20 d;	Hct 0.25–0.40 no significant impact on accuracy, Hct ≥ 0.45 significant impact on accuracy
	N‐desmethyl tamoxifen				15–600	Low, 81.5 Mid, 88.5 High, 84.5	−20 °C, 25 °C and 45 °C: 20 d;	
	4‐Hydroxy tamoxifen				0.5–50	Low, 38.1 Mid, 39.4 High, 41.2	−20 °C and 25 °C: 20 d (at 45 °C increase of 38% in conc. at day 2)	
	Z‐endoxifen				1–40	Low, 47.9 Mid, 48.5 High, 46.1	20 °C and 25 °C: 20 d (at 45 °C increase of 38% in conc. at day 2)	
de Wit et al. (2015)	Pazopanib	DBS; Whatman FTA	4 mm punch–15 μL aliquots	Extraction with Is in formic acid and methanol while mixing	100–50.000	NR	Ambient: 75 d;=	Hct 0.20–0.65 no significant impact on assay performance
Antunes, Raymundo, Wagner, et al. (2015)	Imatinib	DBS; Whatman 903	6 mm punch–60 μL aliquots	(Spots cut in half) Extraction with IS in methanol, incubation while shaking	50–4000	91.5 (range 88.8–93.8)	At low and high: −20 °C, 25 °C, and 45 °C: 36 d	Hct 0.25–0.50 no significant impact on assay performance.
Nijenhuis et al. (2014)	Vemurafenib	DBS; Whatman FTA DMPK‐A	3 mm punch–15 μL aliquots	Extraction with methanol/acetonitrile and IS, vortex mixing and shaking.	1.000–100.000	Low, 92.2 (8.5) High, 105.0 (8.6)	Ambient: 163 d	All at low and high: Spot volume 10–30 μL, punch sites and Hct 0.24–0.45: all no significant impact on accuracy and precision.
Jager, Rosing, Schellens, and Beijnen (2014)	Tamoxifen	DBS; Whatman DMPK‐A	6 mm punch–30 μL aliquots	Extraction with IS in methanol, vortex mixing and sonication, evaporation with nitrogen (30 °C).	2.5–250	Low, 102 (<15) High, 91.2 (<15)	At low and high:2–8 °C and 37 °C: 24 h RT: 4 mo	All at low and high: Hct 0.29–0.48, spot volume 20–50 μL; punch sites, all no significant impact on accuracy and precision.
	Z‐endoxifen				0.5–50	Low, 96.6 (<15) High, 93.9 (<15)		
Xu et al. (2012)	Adavosertib	DBS; Whatman DMPK‐A	3 mm punch–40 μL aliquots	(In 96‐well plate) Extraction with 10 mM ammonium acetate, pH 3.0, IS in in 85% acetonitrile, mixing	2–1000	Low, 71 Mid, 81 High, 80	40 °C and 75% RH: 8 d −20 °C: 6 mo RT: 14 mo	Spot volume 30 and 50 μL, punch site, Hct 0.16–0.85 no significant impact on accuracy and precision.
Kralj et al. (2012)	Imatinib	DBS; Agilent DMS cards	8 mm punch–10 μL aliquots	(96‐well filter plate) addition of IS, extraction with 0.1% formic acid in methanol, mixing	50–5000	Low, 97.4 Mid, 94.8 High, 94.5	RT: 28 d −20 °C: 3 d 40 °C: imatinib and nilotinib stable 3d, dasatinib not.	Hct 0.30–0.60 no significant impact on accuracy at low, mid, high.
	Nilotinib				50–5000	Low, 96.1 Mid, 93.5 High, 90.5		
	Dasatinib				2.5–250	Low, 108.4 Mid, 103.5 High, 93.1		
Maggadani et al. (2021)	Tamoxifen	DBS; PerkinElmer 226 VAMS; Mitra®	whole spot cut–20 μL aliquots Volumetric 20 μL	Similar for DBS and VAMS: vortex mixing and sonication with Is in methanol, evaporation with nitrogen	2.5–200	DBS: Low, 86.84 (5.09) Mid, 82.12 (0.85) High, 84.30 (6.42) VAMS: Low, 91.13 (1.37) Mid, 92.02 (0.94) High, 92.54 (1.56)	Both DBS and VAMS at Low and high: Benchtop: 24 h RT: 60 d −20 °C: 24 h 40 °C: 24 h	
	Z‐endoxifen				2.4–40	DBS: Low, 86.53 (3.90) Mid, 89.21 (4.03) High, 78.59 (3.62) VAMS: Low, 88.63 (0.89) Mid, 87.12 (1.07) High, 87.24 (1.60)		
	4‐Hydroxy tamoxifen				1.5–30	DBS: Low, 75.12 (5.27) Mid, 88.32 (9.00) High, 89.03 (3.48) VAMS: Low, 80.48 (2.12) Mid, 79.70 (1.57) High, 88.27 (1.64)		
	N‐desmethyl tamoxifen				2–600	DBS: Low, 80.41 (1.99) Mid, 91.86 (0.70) High, 87.31 (4.57) VAMS: Low, 91.72 (0.85) Mid, 91.92 (1.17) High, 91.77 (0.73)		
Meertens et al. (2024)	Abiraterone	VAMS; Mitra®	Volumetric 10 μL	Extraction with IS and methanol while shaking with small steel balls, evaporation with nitrogen (40 °C).	2–40	Mid, 85.2 (21.4)	RT and 25 °C: 14 d for all analytes 40 °C: 14 d for all analytes, except D4A and norimatinib: 24 h 3 F/T (−20 °C, −70 °C): 7d for all analytes −20 °C: 12 mo for all analytes −70 °C: 12 mo for all analytes except norimatinib and N‐desethyl sunitinib: 9 mo	Hct 0.29–0.54 no significant impact on accuracy and precision
	D4A				0.5–10	Mid, 69.8 (27.7)		
	Alectinib				100–2000	Mid, 76.2 (5.2)		
	Alectinib‐M4				50–1000	Mid, 79.9 (3.2)		
	Cabozantinib				100–2000	Mid, 99.7 (2.2)		
	Imatinib				200–4000	Mid, 88.0 (1.5)		
	Norimatinib				50–1000	Mid, 84.6 (10.0)		
	Olaparib				400–8000	Mid, 93.1 (2.5)		
	Sunitinib				5–100	Mid, 64.7 (6.0)		
	N‐desethyl sunitinib				5–100	Mid, 69.6 (1.8)		
Krützmann et al. (2023)	Imatinib	VAMS; Mitra®	Volumetric 20 μL	Incubation with 0.1% formic acid in H_2_0, while mixing at 45 °C. Protein precipitation with ACN with 0.1% formic acid including IS. Refrigeration at −20 °C	50–2500	93 (NR)	RT (23–25 °C): 21 d 45 °C: 14 d	Hct 0.25–0.55 no significant impact accuracy and precision. Extraction yield differed ~ 10% among the Hct values
	Norimatinib				50–2500	91 (NR)		
Verougstraete et al. (2022)	Bosutinib	VAMS; Mitra®	Volumetric 10 μL	Pre‐wetting with water while vortex mixing, sonication with IS in methanol, liquid–liquid extraction with MTBE, evaporation with nitrogen.	5–675	Low, 115 (16.9) High, 116 (8.15)	−20 °C, 4 °C, RT: 1 mo for all analytes, except ibrutinib only 2 w at RT;60 °C: 2 d for all analytes except ibrutinib, which is not stable at 60 °C; 3 F/T cycles: all analytes;	All at low and high: Hct 0.18–0.55 no significant impact on accuracy and precision and recovery, except for bosutinib and gilteritinib high imprecision of recovery at QC low Hct 0.18
	Dasatinib				0.5–450	Low, 125% (8.81) High, 118% (4.26)		
	Gilteritinib				25–675	Low, 118% (22.8) High, 104 (7.49)		
	Ibrutinib				5–675	Low, 83 (8.84) High, 104 (6.38)		
	Imatinib				10–2250	Low, 109 (7.23) High, 114 (6.38)		
	Midostaurin				30–2250	Low, 106 (4.39) High, 92 (5.92)		
	Nilotinib				10–2250	Low, 110 (11.6) High, 115 (4.38)		
	Ponatinib				1–450	Low, 114 (4.83) High, 112 (6.02)		
Opitz et al. (2022)	Axitinib	VAMS; Mitra®	Volumetric 20 μL	Pre‐wetting with water, extraction with IS in acetonitrile while shaking.	0.04–10 and 0.5–124	Not executed as sample preparation was similar to Zimmermann et al and Aghai et al, with recovery between 90 and 110%.	RT: 7 d 50 °C: 24 h	NA
Zimmermann et al. (2022)	Afatinib	VAMS; Mitra®	Volumetric 20 μL	Rehydration with water, extraction with IS in acetonitrile while vortex mixing, evaporation.	2–500	Low, 79.9 (4.1) Mid, 81.5 (5.1) High, 79.6 (4.0)	RT (19% RH): 6w for all analytes 60 °C (10% RH): 48 h for all analytes, except afatinib and osimertinib	Low, mid, high: Hct 0.30–0.50 no significant impact on accuracy and precision.
	Axitinib				2–500	Low, 99.4 (6.8) Mid, 100.8 (5.0) High, 101.4 (3.6)		
	Bosutinib				2–500	Low, 91.8 (4.1) Mid, 94.2 (4.5) High, 94.2 (3.7)		
	Cabozantinib				6–1500	Low, 106.3 (3.2) Mid, 101.7 (2.8) High, 107.9 (0.2)		
	Dabrafenib				6–1500	Low, 112.7 (3.5) Mid, 114.1 (2.5) High, 110.0 (2.0)		
	Lenvatinib				2–500	Low, 93.0 (3.7) Mid, 93.6 (3.2) High, 94.4 (2.3)		
	Nilotinib				6–1500	Low, 98.2 (4.6) Mid, 98.2 (3.9) High, 98.2 (2.8)		
	Osimertinib				6–1500	Low, 68.1 (3.2) Mid, 67.7 (3.7) High, 68.9 (7.9)		
	Ruxolitinib				2–500	Low, 99.5 (3.5) Mid, 98.3 (2.8) High, 99.1 2.8)		
	Trametinib				2–500	Low, 98.7 (9.4) Mid, 99.6 (6.1) High, 98.8 (2.7)		
Voggu (2020)	Selumetinib	VAMS: Mitra®	Volumetric 10 μL	(In 96‐well plate) Extraction via and vortex mixing with 0.1% ammoniumhydroxide in methanol and IS working solution. Evaporation with nitrogen at 45 °C.	2.00–2000	Low, 104.1 Mid, 101.6 High, 103.4	At low and high: Bench top RT: 126 d Freezer (−10 to −30 °C): 378 d	NR
Verheijen et al. (2019)	Everolimus	VAMS; Mitra®	Volumetric 10 μL	Extraction with IS and methanol while vortex mixing, ultrasonication and shaking, evaporation with nitrogen.	2.5–100	Low, 23.1 (7.3) High, 20.2 (9.8)	At low and high: ambient: 362 d	At low, mid, and high: Hct 0.30–0.50 significant impact accuracy. Relative biases ranging from −20 to 31%
Rehnmark et al. (2022)	Tamoxifen	Volumetric capillary; Rhelise™ kit	Volumetric 50 μL	Protein precipitation with IS and 0.1% formic acid and acetonitrile while vortex mixing, evaporation with nitrogen	0.1–200	NA	8 ± 2 °C: 14 dRT (20 ± 5 °C): 7 d	NA
	4‐Hydroxy tamoxifen				0.1–200		6–25 °C: 14 d	
	Z‐endoxifen				0.1–200		6–25 °C: 14 d	
Saito et al. (2022)	Lenvatinib	Volumetric capillary; Microsampling Wing	Volumetric 23 μL whole blood for 5.6 μL plasma	Centrifugation of MSW in tube. Snapping of plasma zone, centrifuging, resulting in 5.6 μL plasma. Protein precipitation with IS in acetonitrile while vortex mixing.	1–200	NA	25 °C and 4 °C: 30 d	NA

Abbreviations: d, days; DBS, dried blood spots; Hct, hematocrit; IS, internal standard; Low Mid High, quality control levels; mo, months; NA, not applicable; NR, not reported; RH, relative humidity; RT, room temperature; VAMS, volumetric absorptive microsampling; w, weeks.

**TABLE 2:** Clinical validation of chromatographic mass spectrometric methods using home‐sampling devices to support TDM.

Author (year)	Drugs	Device	Number of samples and patients	Conversion method	Predictive performance conversion method	Remarks
Venkatesh et al. (2023)	Osimertinib, metabolites (AZ5104 and AZ7550)	DBS; HemaPEN	15 plasma and DBS samples from 15 patients	No conversion needed plasma ≈ DBS	NA	
Canil et al. (2023)	Niraparib	Hemaxis DB10	43 plasma and DBS samples from 21 patients	Empirical: conversion factor	Conversion factor met the criteria.	Limitation: Hct must be known in time frame close to home‐sampling because must be within (0.29–0.45).
	Olaparib		52 plasma and DBS samples from 16 patients		Conversion factor met the criteria.	
	Rucaparib		16 plasma and DBS samples from 4 patients		Conversion factor met the criteria.	
Zanchetta et al. (2023)	Lenvatinib	DBS; Whatman 31ETCHR and Whatman 903	10 DBS and plasma samples from 4 patients. DBS made from venous blood using pipette.	Only preliminary data (DBS‐to‐plasma ratio and regression) and no conversion method was evaluated	NA	
Mukai et al. (2022)	Bosutinib	DBS; Whatman 903	DBS collected out of venous whole blood with pipette; DBS and plasma from 19 patients	Empirical: conversion factor and Deming‐regression	Both conversion methods met the criteria.	
	Dasatinib		DBS and plasma from 29 patients (3 excluded due to <LLOQ)	Empirical: correction factor and Deming‐regression	Both conversion methods did not meet the criteria.	Not suitable for trough levels of dasatinib (related to toxicity), but with DBS conc. ≥ 7.25 ng/mL, (corresponding to 4.33 ng/mL in plasma) the predictive performance improved, meaning DBS is suitable for top levels (related to efficacy)
	Ibrutinib		DBS and plasma from 2 patients	NA, too few samples	NA	
	Imatinib		DBS and plasma from 9 patients	Empirical: correction factor and Deming‐regression	Both conversion methods met the criteria.	Also correction found by Antunes, Raymundo, Wagner, et al. (2015) tested as external validation, did meet the criteria.
	Nilotinib		DBS and plasma from 22 patients	Empirical: correction factor and Deming‐regression	Correction factor did not meet the criteria; regression formula met the criteria.	When only including samples drawn after peak concentrations, both conversion methods fell within acceptable criteria (only 7 samples)
	Ponatinib		DBS and plasma from 15 patients	Empirical: correction factor and Deming‐regression	Both conversion methods met the criteria	
Zhang et al. (2022)	Anlotinib	DBS; Whatman 903	DBS and plasma samples from 23 patients	Empirical: Deming‐regression	Conversion method met the criteria	
Braal et al. (2021)	Ribociclib	DBS; Whatman 903	DBS and plasma samples from 17 patients	No conversion method needed		
Poetto et al. (2021)	Palbociclib	DBS; Whatman ET31CHR	DBS capillary and prepared from venous blood, and plasma samples *n* = 20, from 13 patients	Individual Hct normalization with plasma fraction Individual Hct normalization with red blood cell to plasma partitioning coefficient Empirical: correction factor	All methods met the criteria; but drug distribution model was selected as conversion method.	No significant difference between venous and capillary DBS
	Ribociclib		DBS capillary and prepared from venous blood, and plasma samples *n* = 22 from 5 patients		All method met the criteria; correction factor was selected as best method.	No significant difference between venous and capillary DBS
	Letrozol		DBS capillary and prepared from venous blood, and plasma samples *n* = 34 from 11 patients		All method met the criteria; correction factor was selected as best conversion method.	No significant difference between venous and capillary DBS.
Guo et al. (2021)	Bortezomib	DBS Whatman 903	13 DBS and plasma sample pairs from 4 patients	No conversion method needed	NA	
Dillenburg Weiss et al. (2021)	Abiraterone	DBS; Whatman 903	16 DBS (capillary and venous) and plasma sample pairs from 10 patients	Individual Hct normalization with plasma fraction	The conversion method met the criteria.	No significant difference between venous and capillary DBS
	Delta(4)‐abiraterone			Individual Hct normalization with plasma fraction	The conversion method met the criteria.	No significant difference between venous and capillary DBS
Lee et al. (2020)	Radotinib	DBS; Whatman 903 protein saver	DBS and plasma samples from 45 patients	Empirical: Deming regression formula Individual Hct normalization (second‐degree polynomial function)	Both methods met the criteria.	Number of study samples was not sufficient to predict radotinib blood concentrations above 1500 ng/mL.
Iacuzzi et al. (2019)	Imatinib	DBS; Whatman ET31CHR	55 venous and capillary DBS and plasma samples from 26 patients	Individual Hct normalization Empirical: correction factor	Both methods met the criteria. Correction factor showed higher agreement than with Hct normalization. Validation cohort of 12 extra patients with correction factor method met the criteria.	No significant difference between venous and capillary DBS
	Norimatinib				Both methods met the criteria. Correction factor showed higher agreement than with Hct normalization. Validation cohort of 12 extra patients with correction factor method met the criteria.	No significant difference between Venous and capillary DBS
Irie et al. (2018)	Gefitinib	DBS; qualitative filter paper No. 2, 125 g/m^2^ (Advantec)	10 DBS and plasma samples from 10 patients	No conversion method necessary	NA	No influence of Hct tested, as only patients with Hct values within a narrow normal range were included.
Willemsen et al. (2018)	Everolimus	DBS; Whatman 903 protein saver	20 capillary and venous DBS, and venous whole blood samples from 20 patients	Empirical: Passing‐Bablok regression, excluding data of the individual patient from whom the WB concentration is to be predicted. Repeated for each individual patient.	The conversion method met the criteria.	No significant difference between venous and capillary DBS. Without conversion method also good agreement between capillary DBS and whole blood concentrations.
Boons et al. (2017)	Nilotinib	DBS; Whatman DMPK‐C	40 capillary DBS and venipuncture plasma samples from 20 patients	Hct normalization with red blood cell to plasma partitioning coefficient for both individual and population Hct Empirical: Deming regression	All conversion methods met the criteria. Individual Hct did not improve predictive performance compared to population Hct	
Nijenhuis et al. (2016)	Vemurafenib	DBS; Whatman FTA DMPK‐A	41 (duplicate) DBS capillary and plasma samples from 8 patients	Individual Hct normalization with red blood cell to plasma partitioning coefficient Empirical: Deming regression	Both methods met the criteria	
Verheijen et al. (2016)	Pazopanib	DBS; Whatman 903 protein saver	221 paired DBS and plasma samples from 30 patients	Empirical: Deming regression	The conversion method met the criteria.	Hct normalization based conversion did not improve the correlation between calculated and measured plasma (unknown if criteria were met)
Antunes, Raymundo, de Oliveira, et al. (2015)	Tamoxifen	DBS; Whatman 903	DBS and plasma samples from 91 patients	Individual Hct normalization with correction factor	The conversion method met the criteria.	
	N‐desmethyl tamoxifen				The conversion method met the criteria.	
	4‐Hydroxy tamoxifen				The conversion method met the criteria.	
	Z‐endoxifen				The conversion method met the criteria.	
de Wit et al. (2015)	Pazopanib	DBS; Whatman FTA	9 paired DBS venous and plasma samples, and 3 DBS capillary for every patient, 12 patients (so 108 DBS venous and plasma pairs, and 36 capillary paired with venous from 12 patients)	Hct normalization for both individual and population Hct (0.40 for women and 0.45 for men)	Both conversion methods met the criteria	No significant difference between venous and capillary DBS
Antunes, Raymundo, Wagner, et al. (2015)	Imatinib	DBS; Whatman 903	DBS and plasma samples from 50 patients	Individual Hct normalization with plasma factionEmpirical: correction factor	Both conversion methods met the criteria	DBS threshold = 765 ng/mL (converted to 1000 ng/mL in plasma)
Jager, Rosing, Schellens, Beijnen, and Linn (2014)	Tamoxifen	DBS; Whatman FTA DMPK‐A	44 DBS capillary and plasma samples from 44 patients	Population Hct normalization (0.41) and fixed blood cell to plasma partitioning coefficient resulting in empirical correction factor.	Conversion method met the criteria	Empirical formula can be used for Hct between 0.29 and 0.48, Outside these values, an adequate correction can be made by using the equation and computing the patient‐specific Hct.
	Z‐endoxifen				Conversion method met the criteria	
Xu et al. (2012)	Adavosertib	DBS; Whatman DMPK‐A	36 plasma and DBS venous from 12 patients	Only DBS‐to‐plasma ratio and regression were calculated and no conversion method was evaluated	NR	
Kralj et al. (2012)	Imatinib	DBS; Agilent DMS blood spot cards	18 plasma and DBS venous samples from 18 patients	Individual Hct normalization	Conversion method met the criteria	All drugs analyzed together; No capillary DBS used
	Nilotinib		2 plasma and DBS venous samples from 2 patients			
	Dasatinib		3 plasma and DBS venous samples from 3 patients			
Zimmermann et al. (2023)	Cabozantinib	VAMS; Mitra® 20 μL	40 serum and VAMS samples from 18 patients	Empirical: Passing‐Bablok regression Empirical: conversion factor Both individual and population Hct normalization with blood cell‐to‐plasma partitioning	All three conversion methods met the criteria. Individual Hct did not improve predictive performance	
	Dabrafenib		41 serum and VAMS samples from 18 patients		All three conversion methods met the criteria.Individual Hct did not improve predictive performance	
	Nilotinib		41 serum and VAMS samples from 10 patients		All three conversion methods met the criteria. Individual Hct did not improve predictive performance	
	Ruxolitinib		45 serum and VAMS samples from 13 patients		No conversion method met the criteria	
	Trametinib		63 serum and VAMS samples from 17 patients		No conversion method met the criteria	Correlation between VAMS and plasma: *R* ^2^ = 0.5811; in subanalysis showed differences based on gender and obesity
Isberner et al. (2022)	Dabrafenib	VAMS; Mitra® 20 μL	278 serum samples from 27 patients and 169 VAMS samples for dabrafenib from 18 patients, not all paired samples, also VAMS drawn at home	popPK model‐informed VAMS‐to‐serum conversion model involving individual Hct normalization	Conversion factor met the criteria.	
	Trametinib		266 serum samples from 27 patients and 158 VAMS samples from 18 patients not all paired samples, also VAMS drawn at home	popPK model‐informed VAMS‐to‐serum conversion model involving individual Hct normalization with a population value for the blood cell to plasma partition coefficient	Conversion factor met the criteria.	
Verheijen et al. (2019)	Everolimus	VAMS; Mitra®	25 venous whole blood and VAMS capillary samples from 10 patients	Empirical: Deming regression formula	The conversion method met the criteria.	No advantage of VAMS compared to DBS, due to impact of Hct on assay performance. Despite the effect of Hct, using an empirically derived formula, the whole blood everolimus concentration could be back calculated with reasonable accuracy.
Rehnmark et al. (2022)	Tamoxifen	Rhelise™ kit 50 μL	Plasma, venous blood and capillary blood samples from 16 patients	Only preliminary data (DBS to plasma ratio) and no conversion method was evaluated	NA	No significant difference between venous and capillary samples
	4‐Hydroxytamoxifen					Capillary conc. was 56% of the venous concentration
	Z‐endoxifen					Capillary conc. was 545% of venous concentration due to analytical degradation
Saito et al. (2022)	Lenvatinib	MSW (5.6 μL)	35 venous whole blood samples and plasma from 11 patients. MSW was filled from the venous whole blood.	Empirical: Passing‐Bablok regression: No conversion needed MSW plasma ≈ reference plasma	NA	Venous blood was used for comparison with plasma, instead of capillary

Abbreviations: DBS, dried blood spots; Hct, hematocrit; NA, not applicable; NR, not reported; VAMS, volumetric absorptive microsampling.

### 3.2 Analytical Validation Experiments

#### 3.2.1 Analytical Validation Criteria

In order to assess the validity of an analytical method for its purpose, the EMA guidelines or the new ICH M10 guidelines are frequently used as a reference (Capiau et al. 2019; European Medicines Agency 2022). However, only a brief section on dried matrix methods is herein included. It would be advisable to refer to the IATDMCT guideline instead. The IATDMCT's guideline includes an overview of additional evaluation experiments for DBS‐based methods. It is recommended that, as a minimum, recovery, matrix effect, and process efficiency should be tested as additional experiments. These should be reproducible with a %RSD ≤ 15%. This should be conducted at two quality control levels, using samples from six different donors, with one donor modified to three different Hct levels and, all conditions in triplicate. In addition, the volume effect, Hct, and volcano effect should be included as validation experiments, as discussed in this review. For the volume and Hct effect, two quality control levels at three different Hct values were recommended. Similarly for the volcano effect, but in that case for central and peripheral punches. For all criteria, the deviation should not exceed ±15%. Not all articles evaluated these aspects, nor adhered to these strict criteria, as discussed in previous sections.

#### 3.2.2 Sample Volume

The sample volume should be small enough for feasible patient collection while being sufficient to reach clinically relevant lower limit of quantification (LLOQ) values. Reported home‐sampling techniques varied in sample volume (Table 1), with the smallest volume of 2.74 μL analyzed for the DBS Hemapen (Venkatesh et al. 2023). Despite this small volume, no evaporation step was required for concentration, and the clinically relevant LLOQ was still achieved. However, to obtain four 2.74 μL samples, patients still needed to collect 20 μL of blood. Thus, in terms of collection volume, the smallest amount was 10 μL (for DBS and VAMS), which was also the volume used for analysis. For non‐volumetric DBS samples, the smallest punch used was 3 mm, with concomitant aliquots of 10 μL for calibrators and quality control samples. For VAMS, sample volumes of 10 and 20 μL were used with equal frequency. Liquid home‐sampling devices like the Rhelise and MSW collected 50 μL and 23 μL, respectively, with only 5.6 μL analyzed from the MSW (Rehnmark et al. 2022; Saito et al. 2022). Assuming that for 50 μL still only one drop of blood is needed, this will be a feasible volume for patient self‐collection. This has been shown in different studies where feasibility was proved acceptable for volumes up to 20 μL for VAMS and unknown for DBS. In contrast, the Minicollect® device—which was not included in the studies reviewed—required 250–500 μL and resulted in a high rejection rate of 72%. Despite no explicit feasibility study was found for 50 μL – in the specific study a trained nurse obtained the samples—it is expected that this is a manageable self‐collection volume. Generally, 50 μL is widely accepted as the upper limit for defining “microsampling” (Boffel et al. 2024; C.C.L.M. Boons et al. 2019; Van Uytfanghe, Heughebaert, and Stove 2021).

Despite minimizing sample collection volumes to increase likelihood of correct sampling, sensitivity must remain priority. Evaporation steps can be considered to concentrate samples when necessary. Antunes et al. used two DBS disks for sample preparation, meaning at least two spots are needed for one result, with the disadvantage of the lack of back‐up samples (Antunes, Raymundo, de Oliveira, et al. 2015). For axitinib, the LLOQ was lowered from 0.5 to 0.04 ng/mL by using a more sensitive MS/MS detector instead of a single quadrupole detector with a smaller injection volume needed. For performing TDM the LLOQ of 0.5 ng/mL would suffice (Opitz et al. 2022). Other methods reported to optimize source and gas parameters to achieve optimal sensitivity. Overall, all devices provided manageable collection and sample volumes, with sufficient sensitivity.

#### 3.2.3 Analytical Range

Most studies used a wide analytical range between LLOQ and the upper limit of quantification (ULOQ), often 100‐fold or more (Table 1). However, for the home‐sampling approach of TDM, a smaller fit‐for‐purpose range may be sufficient for clinical interpretation, as mainly trough levels will be drawn. The therapeutic target should fall mid‐range, allowing clear differentiation between adequate, toxic, and ineffective concentrations, without requiring an extensive analytical range.

For example, for dasatinib targets for preventing toxicity (trough level ≤2.5 ng/mL and ≤1.5 ng/mL for patients over 50) and to prevent resistance (maximum level >50 ng/mL) suggest that the reported range of 0.5–450 ng/L is more suitable than 2.5–250 or 2–200 ng/mL (Kralj et al. 2012; Mukai et al. 2022; van der Kleij et al. 2023; Verougstraete and Stove 2022). Adjusting the range when transitioning from plasma to whole blood measurements in case of DBS and VAMS may be necessary. Overall, most methods used clinically relevant analytical ranges, with occasionally unnecessarily low LLOQs or high ULOQs.

#### 3.2.4 Sample Preparation and Recovery

For home sampling, dried matrices are most frequently used, such as DBS or VAMS. These matrices present challenges mainly related to sample preparation and resulting analytical recovery, while sample turnaround should meet the clinical needs. For the Rhelise and MSW devices, sample preparation consisted of a simple protein precipitation (Rehnmark et al. 2022; Saito et al. 2022).

Dried sample preparation typically involved extraction and/or protein precipitation using organic solvents (e.g., methanol or acetonitrile), often containing or combined with the internal standard (Table 1). To enhance the extraction, mechanical movement such as vortex mixing and sonication was frequently used, followed by an optional dilution step using a solvent similar to the mobile phase. Organic solvents were preferred for their effectiveness in precipitating proteins and dissolving hydrophobic drugs, but solvent selection is a critical step to ensure robust extraction from DBS or VAMS matrices (Capiau et al. 2019). For instance, the polarity of the analyte may influence affinity for the device compared to the extraction solvent, which was in some cases modified by acid or alkaline additions to the extraction solvent (de Wit et al. 2015; Iacuzzi et al. 2019; Kralj et al. 2012; Krützmann et al. 2023; Verheijen et al. 2016; Voggu et al. 2020; Xu et al. 2012; Zanchetta et al. 2023). The choice of the extraction solvent is important to minimize the influence of Hct on recovery. For instance described by Krützmann et al., accuracy and precision were sufficient with VAMS extraction with methanol, but Hct affected the extraction yield ranging from 105% at HCT 0.25 to 62% at Hct 0.55. Switching to extraction with acetonitrile with 0.1% formic acid resolved this (Krützmann et al. 2023). In one third of the methods, an evaporation step was added in order to concentrate the samples and thereby improve the sensitivity.

Extraction recovery was frequently improved with additional steps, such as pre‐wetting, mechanical movement or physical homogenization. Pre‐wetting is performed by adding water to the dried matrices before adding the organic solvent for extraction. This causes dissolution of blood components, and so effective removal of red blood cells of for instance VAMS tips, increasing recovery of analytes, but also potential causing a greater matrix effect (Xie et al. 2018). This was resolved by Verougstraete et al. by including a liquid–liquid extraction step (Verougstraete and Stove 2022; Xie et al. 2018). Other strategies such as sonication, which was used in one‐third of the DBS and VAMS studies, and physical homogenization with small glass or steel balls were also used in two methods (Meertens et al. 2024; Tré‐Hardy et al. 2016). Sonication not only improved the extraction yield but also proved better reproducibility of the extraction, which probably also counts for physical homogenization (Jager, Rosing, Schellens, and Beijnen 2014; Maggadani et al. 2021; Mano, Kita, and Kusano 2015; Verougstraete and Stove 2022). Sonication, mechanical movement, physical homogenization, and evaporation steps typically lasted between 10 and 90 min.

Extraction recovery was assessed for nearly all DBS and VAMS methods. In Table 1, the sample preparation recovery is reported, representing the fraction of analyte that was successfully extracted, independent of matrix effects. While there are no strict criteria for acceptable recovery levels, higher recovery is generally preferable. Multiple papers referred to a study of Xie et al. who recommend a recovery above 80% to achieve reproducible results during VAMS extraction (Xie et al. 2018). However, it is important that it is both reproducible and sufficient for the analytical method's purpose. In some methods, recovery was variable with %CV values above 15% or even 20% observed (Meertens et al. 2024; Verougstraete and Stove 2022). Interestingly, the lowest recovery found in the literature search was only 20.2% for everolimus in VAMS, but with a %CV of < 10%, indicating that despite the low yield, the recovery is reproducible, which mitigates the issue. Interestingly, everolimus in DBS showed a recovery of 74–81%, while using a similar sample preparation, indicating a clear difference of extractability between devices (Knapen et al. 2018). The clearest differences in recovery between different DBS methods were observed for 4‐hydroxy‐tamoxifen and Z‐endoxifen, where the one method retrieved 40 and 48% from Whatman 903 cards (Antunes, Raymundo, de Oliveira, et al. 2015). Three other methods used either small glass balls during vortexing, or other kind of DBS paper (DMPK‐A and PerkinElmer 226), found recoveries >75% for both metabolites of tamoxifen, thus physical movement or selection of the devices clearly improved the extraction yield (Jager, Rosing, Schellens, and Beijnen 2014; Maggadani et al. 2021; Tré‐Hardy et al. 2016).

Overall, these sample preparation methods are relatively time‐efficient, with techniques like ultrasonication and physical homogenization significantly improving extraction yields, but also lengthening the process. Despite these additional steps, sample analysis can still be completed within a day, maintaining a fast turnaround time.

#### 3.2.5 Stability

Analyte stability from sample collection to analysis is crucial and was primarily assessed at ambient temperature of approximately 20–25 °C. This is based on the assumption that samples are not less stable at lower temperatures. But during transport, especially in situations where samples are exposed to sunlight (e.g., in a mailbox), temperatures can exceed room temperature. Consequently, half of the research groups also tested sample stability at elevated temperatures, ranging from 37 to 60 °C.

In all devices except the MSW, all drugs and metabolites were stable for at least 7 days, and often longer, depending on the maximum period tested. Given the limited stability of abiraterone, bortezomib, ibrutinib, and osimertinib in liquid plasma, the dried forms have improved stability highlighting the practical application of this matrix (Benoist et al. 2017; Guo et al. 2021; Janssen et al. 2019; Rood et al. 2016; van Nuland et al. 2019; van Veelen et al. 2020; Veerman et al. 2019; Verougstraete et al. 2021). For instance, abiraterone is unstable at room temperature in plasma, where in VAMS, it remained stable for 14 days, even at 40 °C, and in DBS minimal 48 h at 60 °C (Benoist et al. 2017; Dillenburg Weiss et al. 2021; Meertens et al. 2024; van Nuland et al. 2019). The MSW demonstrated stability for 5 days, as difficulties arose in centrifuging whole blood to plasma from the capillary samples (Saito et al. 2022). Some studies extended stability testing up to 14 days or even 36 days, while such long stability at high temperature is not necessarily required. Afatinib and osimertinib were not stable in VAMS at 60 °C after 48 h (Zimmermann et al. 2022). Two metabolites of tamoxifen, 4‐hydroxy tamoxifen and Z‐endoxifen, were unstable at 45 °C, showing a 38% increase in concentration after 2 days, possibly due to the degradation of tamoxifen and N‐desmethyl tamoxifen, which are present in much higher concentrations (Antunes, Raymundo, de Oliveira, et al. 2015).

There were also conflicting stability results for dasatinib, ibrutinib, and norimatinib at elevated temperatures. Dasatinib was stable for 72 h in DBS at 40 °C and 48 h in VAMS at 60 ° C, but another study found it unstable for 72 h in DBS at 40 °C (Kralj et al. 2012; Mukai et al. 2022; Verougstraete and Stove 2022). Ibrutinib remained stable for 72 h in DBS at 40 °C but not for 48 h in VAMS (60 °C). Similarly, norimatinib was stable in VAMS at 45 °C for 14 days, but another VAMS study at 40 °C found stability only for 24 h, while also 14 days was tested, although 24 h at 40 °C will suffice in most cases (Krützmann et al. 2023; Meertens et al. 2024). So, even between two dried forms of whole blood, discrepancies may exist, making it essential to test stability for each different application and device. It is recommended to test multiple time points, as this allows for stability data to support shorter intervals, such as 8 or 24 h at elevated temperatures, even if a longer time point, like 72 h proved unstable.

Zimmermann et al. proposed that the instability of afatinib and osimertinib in VAMS at high temperatures might be due to their characteristic as irreversible inhibitors, which could form covalent bonds with the VAMS surface, exacerbated by increased molecular kinetics at elevated temperatures (Zimmermann et al. 2022). Consequently, the observed lower concentrations may be attributed to the lowered extraction recovery, which was observed as instability. Interestingly, this hypothesis may also explain the stability issues observed with ibrutinib, another irreversible kinase inhibitor, which was stable in DBS but not in VAMS. If longer periods of stability at higher temperatures are required, DBS might be preferable over VAMS for irreversible kinase inhibitors, which may be more prone to covalent binding. These inhibitors include afatinib, dacometinib, futibatinib, ibrutinib, mobocertinib, neratinib, osimertinib, and zanubrutinib (P. Y. Lee, Yeoh, and Low 2023; Li et al. 2024). However, this preference is hypothetical, and further research is needed to confirm these findings, as paper cards contain cellulose and therefore hydroxyl‐groups, making them not fully inert (Jager, Rosing, Schellens, and Beijnen 2014).

In conclusion, all analytes were stable at room temperature and the majority remain stable even at elevated temperatures. However, caution is advised for certain drugs, as some may exhibit instability under stress conditions as temperatures above 20–25 °C.

#### 3.2.6 Other Critical Factors Influencing Analytical Performance

Specifically for microsampling techniques, some important factors may have a strong influence on analytical performance, such as Hct and, specifically for DBS methods also spot volume. Hct affects spot size and homogeneity of DBS. Higher Hct levels are related to increased blood viscosity resulting in smaller DBS obtained with the same volume of blood. When using the same punch size to prepare DBS samples, especially with drugs or metabolites that show affinity for red blood cells, Hct variations can seriously affect accuracy (De Kesel et al. 2013; de Vries et al. 2013; Denniff and Spooner 2010).

The VAMS device uses a fixed sample volume, making it potentially unaffected by Hct. However, it was shown that Hct may influence recovery from VAMS and consequently, accuracy and precision (Kok and Fillet 2018). Among the eight VAMS methods reviewed, five studies tested the influence of Hct. For bosutinib and gilteritinib, the variability of the recovery was >15% in case of low concentrations levels in combination with low Hct values of 0.18 (Verougstraete and Stove 2022). Everolimus in VAMS showed a clear Hct‐dependent extraction recovery, with biases from −20% at Hct 0.49 and + 31% at Hct 0.31 (Verheijen et al. 2019). Hypothesis is that the drug or metabolite can bind to proteins, red blood cells, and the polymeric tip, hindering extraction and resulting in lower recovery, with increased effect with higher hydrophobicity of the compound. This is also described as entrapment of the drug or metabolite by red blood cells (De Kesel, Lambert, and Stove 2015; Ye and Gao 2017). Besides Hct influencing the recovery, time can also play a role, as recovery may vary if samples are stored for long periods before analysis. However, this is in most cases not relevant in this context, as the samples will be analyzed shortly after collection and will not be stored for long periods.

For the 23 DBS methods included in this review, 21 assessed the effects of different Hct levels. Among these, four studies identified significant bias related to the Hct levels, due to reduced dispersion of blood caused by higher blood viscosity at higher Hct levels, and vice versa for lower Hct levels (Antunes, Raymundo, de Oliveira, et al. 2015; Dillenburg Weiss et al. 2021; Knapen et al. 2018; Tré‐Hardy et al. 2016). As a consequence, valid calibration was only possible for everolimus in case of Hct ≥0.25, tamoxifen and metabolites with Hct ≤0.40 and for abiraterone and D4A only with Hct ≤0.50, all due to accuracy deviations exceeding ±15%. However, most patients with solid tumors have a less extreme Hct range, reducing the clinical relevance of these findings. Additionally, four other studies observed a positive trend between Hct levels and concentrations, but within ±15% and ≤15% (Guo et al. 2021; Jager, Rosing, Schellens, and Beijnen 2014; Nijenhuis et al. 2014). Thus, an opposite effect in DBS was observed compared to VAMS methods.

Homogeneity of the spot is a DBS specific aspect that should be assessed. The “volcano effect,” where after application of whole blood, red blood cells concentrate more at the center of the spot than at the margins, can impact results (Capiau et al. 2019). Therefore, it is important to test if different punch sites, i.e., central or peripheral, influence the accuracy. Large punches tend to be less affected by homogeneity compared to smaller punches. Among seven DBS studies in this review that assessed different punch sites, none found significant differences in accuracy (Iacuzzi et al. 2019; Jager, Rosing, Schellens, and Beijnen 2014; Nijenhuis et al. 2014; Poetto et al. 2021; Verheijen et al. 2016; Xu et al. 2012; Zhang et al. 2022). However, all experiments used a pipette which will be different than capillary blood from the finger. In one study, using 3 mm DBS punches 3 mm led to underestimation; therefore, 8 mm punches were selected, which cover the entire 10 μL aliquot spot (Zanchetta et al. 2023). The punch‐to‐punch carry‐over was rarely tested, and when it was, it did not result in any issues (Nijenhuis et al. 2014; Verheijen et al. 2016).

Techniques like VAMS, Rhelise, and MSW use a fixed volume of blood unlike DBS cards. Therefore, the influence of the applied volume of blood on DBS cards needs to be evaluated. Despite variability in applied volume (5 to 60 μL), nine DBS studies reported no significant effects on accuracy and precision (Dillenburg Weiss et al. 2021; Iacuzzi et al. 2019; Jager, Rosing, Schellens, and Beijnen 2014; Knapen et al. 2018; Nijenhuis et al. 2014; Poetto et al. 2021; Verheijen et al. 2016; Xu et al. 2012; Zhang et al. 2022). However, larger volume differences possibly lead to more pronounced impacts on accuracy.

To address accuracy issues at certain Hct values, alternative approaches include analyzing the entire DBS, or using a full set of calibration standards that match the Hct of the sample. However, this requires knowledge of the Hct, which is likely not the case in home‐sampling. An empirical approach would be to use the patient's last recorded Hct value. Other methods would involve non‐contact methods as near‐infrared or ultra‐violet/visible spectroscopy‐based analyses, which can estimate Hct, including analyses based on measuring endogenous components such as hemoglobin or potassium content (Capiau et al. 2013; Capiau et al. 2016; Deprez et al. 2023; Velghe, Delahaye, and Stove 2019). Incorporation of Hct in the analytical procedure may improve accuracy and could lead to reasonable accuracy in the clinical application study (Verheijen et al. 2019).

### 3.3 Clinical Validation Experiments

After the analytical validation, clinical validation experiments are required before implementing home‐sampling for TDM in routine care. Of the oral targeted anticancer drugs included in this review, only everolimus is measured in whole blood for routine TDM; the others are typically measured in plasma, both matrices obtained via venipuncture. In the studies published thus far, home‐sampling was mainly performed using whole blood, except for lenvatinib, where the MSW device was used. Out of the 39 articles reviewed, 26 conducted clinical validation experiments.

#### 3.3.1 Capillary Versus Venous Blood

Four studies used venous blood instead of capillary sampling in the clinical validation experiment (Kralj et al. 2012; Mukai et al. 2022; Saito et al. 2022; Zanchetta et al. 2023), an ex vivo setup that can be used if venous and capillary blood concentrations are known to align. Generally, no significant differences are expected between capillary and venous blood for drugs with long half‐lives and if drawn outside the distribution phase (Rowland and Emmons 2010). Two of these studies justified this ex vivo setup with theoretical or literature support, while the others did not. It is recommended to include a comparison of capillary versus venous blood in the clinical validation study design, to ensure that any differences in results are attributed to the microsampling device itself, rather than the sampling site, which is crucial for accurate method validation. Five studies performed this comparison and found no significant differences for pazopanib, everolimus, imatinib and norimatinib, abiraterone and D4A, palbociclib, ribociclib, and letrozole (de Wit et al. 2015; Dillenburg Weiss et al. 2021; Iacuzzi et al. 2019; Poetto et al. 2021; Willemsen et al. 2018). However, tamoxifen's metabolites endoxifen and 4‐hydroxy‐tamoxifen showed significant differences between venous and capillary samples, with capillary concentrations at 545% and 56% of venous levels, respectively. This was probably caused by photo degradation of Z‐endoxifen in the internal standard solution used for the capillary blood quantification (Rehnmark et al. 2022). Therefore, the elevated percentages do not accurately represent true ex vivo levels, but rather result from analytical issues. Since endoxifen is the key metabolite for monitoring, this method using the Rhelise kit is not yet suitable for TDM with home‐sampling.

#### 3.3.2 Conversion Methods

To establish conversion methods from whole blood to plasma concentrations, patient samples were collected from both reference matrices (e.g., plasma) and home‐sampling methods (e.g., DBS or VAMS) to assess the need for conversion. Weighted Deming or Passing‐Bablok regression were used to compare the reference matrix with the capillary samples.

For lenvatinib in the MSW, venous plasma and MSW plasma showed good agreement (Saito et al. 2022). For everolimus, lower concentrations were observed in liquid whole blood compared to DBS and VAMS, despite all matrices involve whole blood (Verheijen et al. 2019; Willemsen et al. 2018). For bortezomib, gefitinib, osimertinib, and ribociclib, whole blood and plasma were considered in accordance with each other, indicating similar distribution between blood cells and plasma (Braal et al. 2021; Guo et al. 2021; Irie et al. 2018; Venkatesh et al. 2023). For most other drugs, regression analyses between plasma and whole blood showed strong correlations, though slopes differed from 1, indicating that conversion methods were necessary. For trametinib in VAMS, the correlation was weaker, with an *R* ^2^ of 0.5811 based on 63 serum and VAMS samples from 17 patients. A subanalysis showed differences based on gender and obesity: Males had an *R* ^2^ of 0.6429 (*n* = 44), females had an *R* ^2^ of 0.5022 (*n* = 19), and non‐obese males showed the strongest correlation with an *R* ^2^ of 0.7581 (*n* = 39).

Various conversion methods were tested across the clinical validations to adjust concentrations between matrices. Iacuzzi et al. reviewed different DBS‐to‐plasma conversion for anticancer drugs, identifying three main approaches (Iacuzzi et al. 2021). First, conversions that consider the individual Hct and the influence of blood‐to‐plasma partitioning. Second, simple normalization based on individual Hct for drugs that do not enter blood cells. Third, conversions based on empirical methods, using correction factors or linear regression formulas (Iacuzzi et al. 2021).

Of the 26 articles with clinical validation experiments, 13 tested conversion methods considered a method based on individual Hct, and 7 of these also evaluated a normalization based on empirical data. Interestingly, the empirical method—requiring no Hct value—met the criteria in all methods and was often the method of preference (Antunes, Raymundo, Wagner, et al. 2015; C. C. L. M. Boons et al. 2017; de Wit et al. 2015; Iacuzzi et al. 2019; Nijenhuis et al. 2016; Poetto et al. 2021; Zimmermann et al. 2023). Only for palbociclib in DBS the drug distribution model was specifically favored as conversion method; however, the simple correction factor also met the criteria (Poetto et al. 2021). For tamoxifen and endoxifen in DBS, a population Hct combined with a fixed partition coefficient effectively resulted in a standardized correction factor (Jager, Rosing, Schellens, Beijnen, and Linn 2014). However, in cases where a patient's Hct fell outside the studied range, the individual Hct method was recommended instead of the empirical method (Jager, Rosing, Schellens, Beijnen, and Linn 2014; Nijenhuis et al. 2016). Also, it was noted that using empirical methods requires limiting the Hct range to the validated values during the analytical validation, which can be a challenge with home‐sampling (Canil et al. 2023). One innovative method involved a popPK model to establish a VAMS‐to‐serum conversion for dabrafenib and trametinib, with also home‐samples informing the model (with lacking of paired plasma samples). Still, Hct values were involved in the obtained formula, complicating its use for home‐sampling (Isberner et al. 2022).

Some conversions did not meet the predefined validation criteria or were only valid under certain conditions. For example, radotinib in DBS did not predict concentrations above 1500 ng/mL due to insufficient sample collection at the higher concentration range, and the predictive performance of dasatinib in DBS was only adequate for plasma concentrations >4.33 ng/mL, i.e., the method can only be applied for the efficacy target of dasatinib (J. Lee et al. 2020; Mukai et al. 2022). For nilotinib in DBS, samples collected during the absorption phase (i.e. 4 h after intake) were excluded in order to fulfill the criteria (Mukai et al. 2022). No successful conversion methods were established for ruxolitinib and trametinib in VAMS, despite testing three approaches (individual and population Hct, correction factor, linear regression) with respectively 45 and 63 sample pairs (Zimmermann et al. 2023). Potentially the short half‐life of ruxolitinib (~ 3 h) with slight differences in time between VAMS and plasma collection and inter‐individual variability in covariates (other than Hct) for trametinib, potentially contributed to these issues (Zimmermann et al. 2023).

For certain drugs, conflicting results were obtained for clinical validation. For ribociclib in DBS, Braal et al. concluded that no conversion was needed (based on 17 paired samples and patients), whereas Poetto et al. found that DBS were approximately a factor 1.4 higher than plasma (based on 22 samples from 5 patients) (Braal et al. 2021; Poetto et al. 2021). Imatinib and tamoxifen were well studied, with at least two articles with sufficient amount of samples and patients researched. Therefore, the conversion methods could be adopted immediately without the need to establish a new conversion method.

#### 3.3.3 Clinical Evaluation Criteria

The established conversion methods were evaluated using several different criteria, including the EMA guideline where conversion experiments can be interpreted as a cross‐validation, where at least two‐thirds of the predicted concentrations must fall within ± 20% (or 1.96 standard deviation) of the reference concentrations, analyzed via Bland–Altman analyses (European Medicines Agency 2011). Also measures such as mean absolute percentage error (MAPE), mean percentage prediction error (MPPE), median prediction error (MPE), and residual mean square error (RMSE) were used, which are particularly convenient for comparing multiple conversion methods. Some methods used a regression between the estimated plasma concentrations and the reference plasma concentrations to check the slope.

A minimum of 40 paired samples has been recommended, covering the full relevant clinical range. A sample size of smaller than 40 is only possible when the %CV of the method is <5% and the range ratio from LLOQ to ULOQ is <25 (Capiau et al. 2019). Ideally, 80 paired samples should be collected—40 to develop the conversion method and 40 to validate the established method. In studies with limited patient numbers available, which will be the case for many oral targeted anticancer drugs, a minimum of 40 samples from 25 unique patients is recommended (Capiau et al. 2019). Six studies met this requirement, all using DBS, for imatinib (2×), pazopanib, radotinib, and tamoxifen (2×). If fewer than 25 patients were available, the jackknife method is recommended, though only one study reported application (Willemsen et al. 2018). Most studies included >20 patients or samples but did not meet the full criteria. However, if the paired samples cover the concentration range and yield a reliable conversion, this can be acceptable. For instance, olaparib in DBS conversion with 52 samples from 16 patients led to 94.2% of the predicted concentrations within 20% of the plasma concentration, while for ribociclib in 17 paired samples no need for conversion was shown.

### 3.4 Practical Recommendations

In recent years, advancements in microsampling techniques have been made and volumetric DBS devices have been introduced as an alternative to the traditional “paper + punch” methods. While these newer devices have not replaced conventional approaches on a large scale yet, they offer various advantages by ensuring accurate blood volume collection and reducing Hct‐related variability. This volumetric aspect of the device diminish the need to test and validate the impact of different Hct ranges on spot size and punch site during analytical validation. Examples of these volumetric DBS devices include Hemapen, Hemaxis DB10, and the Capitainer (Canil et al. 2023; Deprez et al. 2019; Leuthold et al. 2015; Neto et al. 2018; Velghe and Stove 2018; Venkatesh et al. 2023). When developing and validating a home‐sampling method for oral targeted anti‐cancer drugs, it is recommended to consider volumetric DBS or VAMS, or explore even more innovative microsampling methods.

Additionally, the use of sensitive LC‐MS/MS techniques is recommended to improve detection limits and analytical reliability. The selection of an appropriate analytical range is essential, with calibration curves ideally not exceeding two orders of magnitude of analyte concentration to maintain clinical relevance without unnecessary sensitivity. Besides the standard validation parameters that should be investigated, stability and recovery should be extensively tested under various conditions, including different temperatures, concentrations, and time intervals, with particular attention to handling from collection of the sample to receipt at the laboratory. For drugs with established conversion methods, such as imatinib and endoxifen, the previously described methods can be implemented following external validation, making the clinical validation process more efficient. Furthermore, patients should be well instructed to ensure correct self‐sampling, minimizing pre‐analytical errors and improving sample quality.

## 4 Conclusions

Home‐sampling is a promising tool for TDM of oral targeted anticancer drugs, though it presents unique challenges compared to conventional hospital‐based TDM. One of the critical aspects was ensuring robust sample collection and subsequent pretreatment. Stability during transportation, as well as factors like Hct, were carefully studied. For a variety of analytes, the analytical methods reviewed, showed sufficient robustness to support home‐based TDM. Clinical validation studies also reported good results for most analytes, although external validation of established conversion methods would be a crucial future step before widespread clinical implementation. Collaboration across hospitals could facilitate the establishment of home‐sampling infrastructures for TDM, leveraging shared expertise and resources.

After analytical and clinical validation, an essential consideration is the feasibility of self‐sampling by patients. Boffel et al. demonstrated that self‐sampling at home was overall positively received by inexperienced individuals, with success rates for one successful sample ranging from 65 to 89% across different devices such as DBS, VAMS, Tasso‐M20, and Capitainer®. The exception was the Minicollect device, which had a lower success rate of 12%, probably due to large collection volumes. While these results suggest home‐sampling is feasible for many devices, there remains room for improvement (Boffel et al. 2024).

Interestingly, while mainly DBS and VAMS devices were included in this review, plasma separation devices—except for the MSW—were underrepresented in the literature. These devices, such as Telimmune™ separation cards and the Capitainer® SEP10 (currently in development) could offer theoretical advantages allowing direct measurement in (dried) plasma (Bondan and Linden 2023). However, analytical validation experiments should be performed to ensure a robust method, for instance whether Hct influences the separation quality. Also clinical validation experiments must be performed to test if these devices provide consistent results with conventional plasma samples.

In summary, the findings of this review support the implementation of home‐sampling for TDM of oral targeted anticancer drugs. Certain methods, such as those for imatinib and tamoxifen, may be clinically implemented immediately, while others require external validation before or during their implementation. Future research should focus on device‐specific challenges, and ensuring patient feasibility to make home‐sampling a routine clinical practice.

## Author Contributions

**Marinda Meertens:** conceptualization, methodology, investigation, writing – original draft, writing – review and editing. **Hilde Rosing:** conceptualization, supervision, writing – review and editing. **Neeltje Steeghs:** conceptualization, supervision, writing – review and editing. **Jos H. Beijnen:** supervision, writing – review and editing. **Alwin D.R. Huitema:** conceptualization, supervision, writing – review and editing.

## Conflicts of Interest

Neeltje Steeghs provided consultation or attended advisory boards for Boehringer Ingelheim, Ellipses Pharma, GlaxoSmithKline, Incyte, Luszana. N Steeghs received research grants from Abbvie, Actuate Therapeutics, Amgen, Array, Ascendis Pharma, AstraZeneca, Bayer, Blueprint Medicines, Boehringer Ingelheim, Bristol‐Myers Squibb, Cantargia, CellCentric, Cogent Biosciences, Cresecendo Biologics, Cytovation, Deciphera, Dragonfly, Eli Lilly, Exelixis, Genentech, GlaxoSmithKline, IDRx, Immunocore, Incyte, InteRNA, Janssen, Kinnate Biopharma, Kling Biotherapeutics, Lixte, Luszana, Merck, Merck Sharp & DohmeMerck Sharp & Dohme, Merus, Molecular Partners, Navire Pharma, Novartis, Numab Therapeutics, Pfizer, Relay Pharmaceuticals, Revolution Medicin, Roche, Sanofi, Seattle Genetics, Taiho, Takeda. All outside the submitted work, all payment to the Netherlands Cancer Institute. Jos. H. Beijnen is (indirect) shareholder of Modra Pharmaceuticals B.V. He is (partly) patent holder of oral taxane formulations which are clinically developed by Modra Pharmaceuticals B.V. (a spinoff company of the Netherlands Cancer Institute, not related to this work). Marinda Meertens, Hilde Rosing, Neeltje Steeghs, Jos H. Beijnen, and Alwin D.R. Huitema declare that the research was conducted in the absence of any commercial or financial relationships that could be construed as a potential conflict of interest.

## Supporting information


**Data S1.** Supporting Information
