# Supplementary material for: Advancing Therapeutic Drug Monitoring for Oral Targeted Anticancer Drugs: From Hospital‐Based Towards Home‐Sampling
Source: Biomed Chromatogr. 2025 Mar 14;39(5):e70056. doi: 10.1002/bmc.70056 (PMC11907759; doi:10.1002/bmc.70056)
Supplement: Supplementary file 1 — Data S1. Supporting Information [file BMC-39-e70056-s001.pdf]

Supplementary material - searches

Medline via Ovid:

Ovid MEDLINE(R) ALL <1946 to February 06, 2024>		
**#**	**Query**	**Hits**
1	"dried blood spot testing"/ or (exp fingers/ and exp "Blood Specimen Collection"/)	2295
2	("dried blood spot?" or DBS or "dried plasma spot?" or DPS or microsampl* or VAMS or "plasma separation card" or "finger prick" or "finger stick").ti,ab,kf.	23870
3	((home or self) adj3 sampl*).ti,ab,kf.	6676
4	((finger* or digit?) adj3 (sampl* or blood)).ti,ab,kf.	4910
5	(capillary adj3 (blood or plasma or sampl*)).ti,ab,kf.	12257
6	or/1-5 [dbs]	45490
7	Afatinib/ or Axitinib/ or Crizotinib/ or Dasatinib/ or "Erlotinib Hydrochloride"/ or Everolimus/ or "Imatinib Mesylate"/ or lapatinib/ or exp Sirolimus/ or Sorafenib/ or Sunitinib/ or Vemurafenib/ or exp Tamoxifen/	79262
8	(adagrasib or abemaciclib or acalabrutinib or alectinib or Alpelisib or asciminib or avapritinib or binimetinib or bosutinib or brigatinib or cabozantinib or capmatinib or ceritinib or cobimetinib or dabrafenib or dacomitinib or duvelisib or encorafenib or entrectinib or fedratinib or futibatinib or gefitinib or gilteritinib or glasdegib or ibrutinib or idelalisib or ivosidenib or larotrectinib or lenvatinib or lorlatinib or midostaurin or neratinib or nilotinib or nintedanib or niraparib or olaparib or osimertinib or palbociclib or pazopanib or pemigatinib or pirtobrutinib or ponatinib or pralsetinib or quizartinib or regorafenib or ribociclib or ripretinib or ruxolitinib or selpercatinib or "AZD 6244" or sonidegib or sotorasib or talazoparib or temsirolimus or tepotinib or tivozanib or trametinib or tucatinib or vandetanib or venetoclax or HhAntag691 or zanubrutinib or abiraterone or enzalutamide).rn.	30941
9	(adagrasib or abemaciclib or Verzenio or acalabrutinib or Calquence or Afatinib or Gilotrif or alectinib or Alecensa or Alpelisib or Piqray or asciminib or Scemblix or avapritinib or ayvakit or Axitinib or Inlyta or binimetinib or Mektovi or bosutinib or brigatinib or Alunbrig or cabozantinib or Cometriq or capmatinib or Tabrecta or ceritinib or Zykadia or cobimetinib or Cotellic or Crizotinib or Xalkori or dabrafenib or dacomitinib or Vizimpro or Dasatinib or Sprycel or duvelisib or Copiktra or encorafenib or Braftovi or entrectinib or Rozlytrek or Erlotinib or Tarceva or Everolimus or Certican or Zortress or Afinitor or fedratinib or Inrebic or futibatinib or gefitinib or Iressa or gilteritinib or Xospata or glasdegib or Daurismo or ibrutinib or Imbruvica or idelalisib or Zydelig or Imatinib or Gleevec or Glivec or ivosidenib or Tibsovo or lapatinib or Tykerb or larotrectinib or Vitrakvi or lenvatinib or Lenvima or lorlatinib or Lorbrena or midostaurin or Rydapt or neratinib or Nerlynx or nilotinib or Tasigna or nintedanib or Ofev or Vargatef or niraparib or Zejula or olaparib or Lynparza or osimertinib or mereletinib or Tagrisso or palbociclib or Ibrance or pazopanib or Votrient or pemigatinib or Pemazyre or pirtobrutinib or Jaypirca or ponatinib or Iclusig or pralsetinib or gavreto or quizartinib or regorafenib or Stivarga or ribociclib or Kisqali or ripretinib or rucaparib or Rubraca or ruxolitinib or Jakavi or Jakafi or opzelura or selpercatinib or "AZD 6244" or AZD6244 or selumetinib or Sirolimus or Rapamycin or Rapamune or sonidegib or Odomzo or Sorafenib or Nexavar or sotorasib or lumakras or Sunitinib or Sutent or talazoparib or Talzenna or temsirolimus or Torisel or tepotinib or tepmetko or tivozanib or fotivda or trametinib or tucatinib or irbinitinib or tukysa or vandetanib or Caprelsa or Zactima or Vemurafenib or Zelboraf or venetoclax or Venclexta or HhAntag691 or vismodegib or erivedge or zanubrutinib or Brukinsa or abiraterone or Tamoxifen or Nolvadex or Novaldex or Tomaxithen or Zitazonium or Soltamox or Raloxifene or Keoxifene or Evista or Toremifene or Fareston or enzalutamide or Xtandi).ti,ab,kf.	169417

10	or/7-9 [medicals]	185453
11	exp "protein kinase inhibitors"/	123849
12	"protein kinase inhibitors".rn.	59318
13	"protein kinase inhibitor?".ti,ab,kf.	5625
14	or/11-13 [PKI]	127466
15	"Tyrosine Kinase Inhibitors"/	500
16	("tyrosine kinase inhibitor?" or "tyrosine protein kinase inhibitor?").ti,ab,kf.	39138
17	or/15-16 [TKI]	39154
18	6 and (10 or 14 or 17)	163

Embase.com

#	Query	Hits
1	'dried blood spot testing'/exp OR 'dried plasma spot'/de OR 'volumetric absorptive microsampling'/de OR ('finger'/exp AND 'blood sampling'/exp)	7102
2	('dried blood spot$' OR DBS OR 'dried plasma spot$' OR DPS OR microsampl* OR 'micro sampl*' OR VAMS OR 'plasma separation card' OR 'finger prick$' OR 'finger stick$'):ti,ab,kw	39026
3	((home OR self) NEAR/3 sampl*):ti,ab,kw	8573
4	((finger* OR digit$) NEAR/3 (sampl* OR blood)):ti,ab,kw	6772
5	(capillary NEAR/3 (blood OR plasma OR sampl*)):ti,ab,kw	17099
6	#1 OR #2 OR #3 OR #4 OR #5	69413
7	'adagrasib'/exp OR 'abemaciclib'/exp OR 'acalabrutinib'/exp OR 'afatinib'/exp OR 'alectinib'/exp OR 'alpelisib'/exp OR 'asciminib'/exp OR 'avapritinib'/exp OR 'axitinib'/exp OR 'binimetinib'/exp OR 'bosutinib'/exp OR 'brigatinib'/exp OR 'cabozantinib'/exp OR 'capmatinib'/exp OR 'ceritinib'/exp OR 'cobimetinib'/exp OR 'crizotinib'/exp OR 'dabrafenib'/exp OR 'dacomitinib'/exp OR 'dasatinib'/exp OR 'duvelisib'/exp OR 'encorafenib'/exp OR 'entrectinib'/exp OR 'erlotinib'/exp OR 'everolimus'/exp OR 'fedratinib'/exp OR 'futibatinib'/exp OR 'gefitinib'/exp OR 'gilteritinib'/exp OR 'glasdegib'/exp OR 'ibrutinib'/exp OR 'idelalisib'/exp OR 'imatinib'/exp OR 'ivosidenib'/exp OR 'lapatinib'/exp OR 'larotrectinib'/exp OR 'lenvatinib'/exp OR 'lorlatinib'/exp OR 'midostaurin'/exp OR 'neratinib'/exp OR 'nilotinib'/exp OR 'nintedanib'/exp OR 'niraparib'/exp OR 'olaparib'/exp OR 'osimertinib'/exp OR 'palbociclib'/exp OR 'pazopanib'/exp OR 'pemigatinib'/exp OR 'pirtobrutinib'/exp OR 'ponatinib'/exp OR 'pralsetinib'/exp OR 'quizartinib'/exp OR 'regorafenib'/exp OR 'ribociclib'/exp OR 'ripretinib'/exp OR 'rucaparib'/exp OR 'ruxolitinib'/exp OR 'selpercatinib'/exp OR 'selumetinib'/exp OR 'sirolimus'/exp OR 'sonidegib'/exp OR 'sorafenib'/exp OR 'sotorasib'/exp OR 'sunitinib'/exp OR 'talazoparib'/exp OR 'temsirolimus'/exp OR 'tepotinib'/exp OR 'tivozanib'/exp OR 'trametinib'/exp OR 'tucatinib'/exp OR 'vandetanib'/exp OR 'vemurafenib'/exp OR 'venetoclax'/exp OR 'vismodegib'/exp OR 'zanubrutinib'/exp OR 'abiraterone'/exp OR 'tamoxifen'/exp OR 'enzalutamide'/exp	414660
8	(adagrasib OR abemaciclib OR Verzenio OR acalabrutinib OR Calquence OR Afatinib OR Gilotrif OR alectinib OR Alecensa OR Alpelisib OR Piqray OR asciminib OR Scemblix OR avapritinib OR ayvakit OR Axitinib OR Inlyta OR binimetinib OR Mektovi OR bosutinib OR brigatinib OR Alunbrig OR cabozantinib OR Cometriq OR capmatinib OR Tabrecta OR ceritinib OR Zykadia OR cobimetinib OR Cotellic OR Crizotinib OR Xalkori OR dabrafenib OR dacomitinib OR Vizimpro OR Dasatinib OR Sprycel OR duvelisib OR Copiktra OR encorafenib OR Braftovi OR entrectinib OR Rozlytrek OR Erlotinib OR Tarceva OR Everolimus OR Certican OR Zortress OR Afinitor OR fedratinib OR Inrebic OR futibatinib OR gefitinib OR Iressa OR gilteritinib OR Xospata OR glasdegib OR Daurismo OR ibrutinib OR Imbruvica OR idelalisib OR Zydelig OR Imatinib OR Gleevec OR Glivec OR ivosidenib OR Tibsovo OR lapatinib OR Tykerb OR larotrectinib OR Vitrakvi OR lenvatinib OR Lenvima OR	291777

	lorlatinib OR Lorbrena OR midostaurin OR Rydapt OR neratinib OR Nerlynx OR nilotinib OR Tasigna OR nintedanib OR Ofev OR Vargatef OR niraparib OR Zejula OR olaparib OR Lynparza OR osimertinib OR mereletinib OR Tagrisso OR palbociclib OR Ibrance OR pazopanib OR Votrient OR pemigatinib OR Pemazyre OR pirtobrutinib OR Jaypirca OR ponatinib OR Iclusig OR pralsetinib OR gavreto OR quizartinib OR regorafenib OR Stivarga OR ribociclib OR Kisqali OR ripretinib OR rucaparib OR Rubraca OR ruxolitinib OR Jakavi OR Jakafi OR opzelura OR selpercatinib OR 'AZD 6244' OR AZD6244 OR selumetinib OR Sirolimus OR Rapamycin OR Rapamune OR sonidegib OR Odomzo OR Sorafenib OR Nexavar OR sotorasib OR lumakras OR Sunitinib OR Sutent OR talazoparib OR Talzenna OR temsirolimus OR Torisel OR tepotinib OR tepmetko OR tivozanib OR fotivda OR trametinib OR tucatinib OR irbinitinib OR tukysa OR vandetanib OR Caprelsa OR Zactima OR Vemurafenib OR Zelboraf OR venetoclax OR Venclexta OR HhAntag691 OR vismodegib OR erivedge OR zanubrutinib OR Brukinsa OR abiraterone OR Tamoxifen OR Nolvadex OR Novaldex OR Tomaxithen OR Zitazonium OR Soltamox OR Raloxifene OR Keoxifene OR Evista OR Toremifene OR Fareston OR enzalutamide OR Xtandi):ti,ab,kw	
9	#7 OR #8	456597
10	'protein kinase inhibitor'/exp	747737
11	'protein kinase inhibitor$':ti,ab,kw	6678
12	#10 OR #11	749130
13	'tyrosine kinase inhibitor$':ti,ab,kw OR 'tyrosine protein kinase inhibitor$':ti,ab,kw	63948
14	'protein tyrosine kinase inhibitor'/exp	426813
15	#13 OR #14	432585
16	#6 AND (#9 OR #12 OR #15)	748
17	#16 NOT ('conference abstract'/it OR 'conference review'/it)	590

Scopus

**Query**	**Hits**
(<br>TITLE-ABS(("dried blood spot*" OR {DBS} OR "dried plasma spot*" OR {DPS} OR microsampl* OR "micro sampl*" OR {VAMS} OR "plasma separation card" OR "finger prick*" OR "finger stick*") OR ((home OR self) W/2 sampl*) OR ((finger* OR digit*) W/2 (sample* OR blood)) OR (capillary W/2 (blood OR plasma OR sampl*)))<br>OR AUTHKEY(("dried blood spot*" OR {DBS} OR "dried plasma spot*" OR {DPS} OR microsampl* OR "micro sampl*" OR {VAMS} OR "plasma separation card" OR "finger prick*" OR "finger stick*") OR ((home OR self) W/2 sampl*) OR ((finger* OR digit*) W/2 (sample* OR blood)) OR (capillary W/2 (blood OR plasma OR sampl*)))<br>)<br>AND (<br>TITLE-ABS(adagrasib OR abemaciclib OR Verzenio OR acalabrutinib OR Calquence OR Afatinib OR Gilotrif OR alectinib OR Alecensa OR Alpelisib OR Piqray OR asciminib OR Scemblix OR avapritinib OR ayvakit OR Axitinib OR Inlyta OR binimetinib OR Mektovi OR bosutinib OR brigatinib OR Alunbrig OR cabozantinib OR Cometriq OR capmatinib OR Tabrecta OR ceritinib OR Zykadia OR cobimetinib OR Cotellic OR Crizotinib OR Xalkori OR dabrafenib OR dacomitinib OR Vizimpro OR Dasatinib OR Sprycel OR duvelisib OR Copiktra OR encorafenib OR Braftovi OR entrectinib OR Rozlytrek OR Erlotinib OR Tarceva OR Everolimus OR Certican OR Zortress OR Afinitor OR fedratinib OR Inrebic OR futibatinib OR gefitinib OR Iressa OR gilteritinib OR Xospata OR glasdegib OR Daurismo OR ibrutinib OR Imbruvica OR idelalisib OR Zydelig OR Imatinib OR Gleevec OR Glivec OR ivosidenib OR Tibsovo OR lapatinib OR Tykerb OR larotrectinib OR Vitrakvi OR lenvatinib OR Lenvima OR lorlatinib OR Lorbrena OR midostaurin OR Rydapt OR neratinib OR Nerlynx OR nilotinib OR	187

Tasigna OR nintedanib OR Ofev OR Vargatef OR niraparib OR Zejula OR olaparib OR Lynparza OR osimertinib OR mereletinib OR Tagrisso OR palbociclib OR Ibrance OR pazopanib OR Votrient OR pemigatinib OR Pemazyre OR pirtobrutinib OR Jaypirca OR ponatinib OR Iclusig OR pralsetinib OR gavreto OR quizartinib OR regorafenib OR Stivarga OR ribociclib OR Kisqali OR ripretinib OR rucaparib OR Rubraca OR ruxolitinib OR Jakavi OR Jakafi OR opzelura OR selpercatinib OR "AZD 6244" OR AZD6244 OR selumetinib OR Sirolimus OR Rapamycin OR Rapamune OR sonidegib OR Odomzo OR Sorafenib OR Nexavar OR sotorasib OR lumakras OR Sunitinib OR Sutent OR talazoparib OR Talzenna OR temsirolimus OR Torisel OR tepotinib OR tepmetko OR tivozanib OR fotivda OR trametinib OR tucatinib OR irbinitinib OR tukysa OR vandetanib OR Caprelsa OR Zactima OR Vemurafenib OR Zelboraf OR venetoclax OR Venclexta OR HhAntag691 OR vismodegib OR erivedge OR zanubrutinib OR Brukinsa OR abiraterone OR Tamoxifen OR Nolvadex OR Novaldex OR Tomaxithen OR Zitazonium OR Soltamox OR Raloxifene OR Keoxifene OR Evista OR Toremifene OR Fareston OR enzalutamide OR Xtandi)<br>OR AUTHKEY(adagrasib OR abemaciclib OR Verzenio OR acalabrutinib OR Calquence OR Afatinib OR Gilotrif OR alectinib OR Alecensa OR Alpelisib OR Piqray OR asciminib OR Scemblix OR avapritinib OR ayvakit OR Axitinib OR Inlyta OR binimetinib OR Mektovi OR bosutinib OR brigatinib OR Alunbrig OR cabozantinib OR Cometriq OR capmatinib OR Tabrecta OR ceritinib OR Zykadia OR cobimetinib OR Cotellic OR Crizotinib OR Xalkori OR dabrafenib OR dacomitinib OR Vizimpro OR Dasatinib OR Sprycel OR duvelisib OR Copiktra OR encorafenib OR Braftovi OR entrectinib OR Rozlytrek OR Erlotinib OR Tarceva OR Everolimus OR Certican OR Zortress OR Afinitor OR fedratinib OR Inrebic OR futibatinib OR gefitinib OR Iressa OR gilteritinib OR Xospata OR glasdegib OR Daurismo OR ibrutinib OR Imbruvica OR idelalisib OR Zydelig OR Imatinib OR Gleevec OR Glivec OR ivosidenib OR Tibsovo OR lapatinib OR Tykerb OR larotrectinib OR Vitrakvi OR lenvatinib OR Lenvima OR lorlatinib OR Lorbrena OR midostaurin OR Rydapt OR neratinib OR Nerlynx OR nilotinib OR Tasigna OR nintedanib OR Ofev OR Vargatef OR niraparib OR Zejula OR olaparib OR Lynparza OR osimertinib OR mereletinib OR Tagrisso OR palbociclib OR Ibrance OR pazopanib OR Votrient OR pemigatinib OR Pemazyre OR pirtobrutinib OR Jaypirca OR ponatinib OR Iclusig OR pralsetinib OR gavreto OR quizartinib OR regorafenib OR Stivarga OR ribociclib OR Kisqali OR ripretinib OR rucaparib OR Rubraca OR ruxolitinib OR Jakavi OR Jakafi OR opzelura OR selpercatinib OR "AZD 6244" OR AZD6244 OR selumetinib OR Sirolimus OR Rapamycin OR Rapamune OR sonidegib OR Odomzo OR Sorafenib OR Nexavar OR sotorasib OR lumakras OR Sunitinib OR Sutent OR talazoparib OR Talzenna OR temsirolimus OR Torisel OR tepotinib OR tepmetko OR tivozanib OR fotivda OR trametinib OR tucatinib OR irbinitinib OR tukysa OR vandetanib OR Caprelsa OR Zactima OR Vemurafenib OR Zelboraf OR venetoclax OR Venclexta OR HhAntag691 OR vismodegib OR erivedge OR zanubrutinib OR Brukinsa OR abiraterone OR Tamoxifen OR Nolvadex OR Novaldex OR Tomaxithen OR Zitazonium OR Soltamox OR Raloxifene OR Keoxifene OR Evista OR Toremifene OR Fareston OR enzalutamide OR Xtandi)<br>OR TITLE-ABS("protein kinase inhibitor*" OR "tyrosine kinase inhibitor*" OR "tyrosine protein kinase inhibitor*")<br>OR AUTHKEY("protein kinase inhibitor*" OR "tyrosine kinase inhibitor*" OR "tyrosine protein kinase inhibitor*")<br>)	
